# Self-reported physical activity behavior of a multi-ethnic adult population within the urban and rural setting in Suriname

**DOI:** 10.1186/s12889-015-1807-1

**Published:** 2015-05-12

**Authors:** Se-Sergio M Baldew, Ingrid SK Krishnadath, Christel CF Smits, Jerry R Toelsie, Luc Vanhees, Veronique Cornelissen

**Affiliations:** Department of Physical Therapy, Faculty of Medical Sciences, Anton de Kom University of Suriname, Paramaribo, Suriname; Department of Rehabilitation Sciences, Research Center for Cardiovascular Rehabilitation, Faculty of Kinesiology and Rehabilitation Sciences, Katholieke Universiteit Leuven, Leuven, Belgium; Department of Public Health, Faculty of Medical Sciences, Anton de Kom University of Suriname, Paramaribo, Suriname; Department of Physiology, Faculty of Medical Sciences, Anton de Kom University of Suriname, Paramaribo, Suriname

**Keywords:** Self-reported physical activity, Multi-ethnic population, WHO STEPS, Global physical activity questionnaire

## Abstract

**Background:**

Physical activity (PA) plays an important role in the combat against noncommunicable diseases including cardiovascular diseases. In order to develop appropriate PA intervention programs, there is a need to evaluate PA behavior. So far, there are no published data on PA available for Suriname. Therefore, we aim to describe PA behavior among the multi-ethnic population living in urban and rural areas of Suriname.

**Methods:**

The World Health Organization (WHO) STEPwise approach to chronic disease risk factor surveillance (STEPS) was conducted in a national representative sample (N = 5751; 48.6% men) aged 15–64 years between March and September 2013. Physical activity data were assessed using the Global physical activity questionnaire (GPAQ) and analyzed according to the GPAQ guidelines. The prevalence of meeting the recommended PA level and prevalence ratios (PR) were computed.

**Results:**

Only 55.5% of the overall population met the WHO recommended PA levels (urban coastal area: 55.7%, rural coastal area: 57.9%, rural interior area: 49.1%). Women were less likely to meet the recommended PA level (49% vs 62.4%; p < 0.0001) and with increasing age the PR for recommended level of PA decreased (p < 0.0001). Compared to the Hindustani’s, the largest ethnic group, the Javanese reported the lowest percentage of people meeting recommended PA level (PR = 0.92; p = 0.07).

**Conclusion:**

Around half of the population meets the recommended PA level. Future lifestyle interventions aiming at increasing PA should especially focus on women and older individuals as they are less likely to meet the recommended levels of PA.

## Background

Cardiovascular diseases (CVD) remain the number one cause of death globally, with over 80% of these deaths reported in low and middle income countries [1]. In prevention of cardiovascular and other noncommunicable diseases (NCD), physical activity (PA) plays an important role [2-4]. In contrast to what is known about PA in Europe [5,6] and North America [7] there are no published PA data available for Suriname. However, 27% of all deaths in Suriname are caused by cardiovascular diseases, making them the number one cause of mortality [8].

There is an obvious need for an adequate evaluation of PA behavior in order to initiate and implement appropriate PA intervention programs in Suriname. Since Suriname has a diverse ethnic population and the prevalence of CVD and risk behaviors associated with these diseases has been shown to differ among ethnic groups [9-11], evaluation of PA behavior across the ethnic groups needs to be taken into account.

In addition to the large variability between countries with regard to the proportion of individuals meeting the recommended PA level [12,13], it has also been shown that these levels might differ within a country depending on the area [13,14]. In general, individuals living in urban areas tend to be less active compared to those living in rural areas [14-18], although one report concluded the opposite [19].

Therefore, if we aim to develop effective interventions to increase PA behavior, it is important to take all these differences, i.e. ethnicity and area into account. Thus, the main objective of this study was to describe PA behavior in a representative sample of the Surinamese population, across urban and rural areas. Secondly, we aimed to investigate whether there are differences in PA among the different ethnic groups.

## Methods

This cross-sectional study was based on the WHO STEPwise approach to Chronic Disease Risk Factor Surveillance (STEPS) protocol. A detailed description of the study design and participant recruitment has been reported elsewhere [20]. Briefly, a sample size was calculated based on area, gender distribution and on five age groups between 15 and 64 years. Furthermore, ethnic groups were taken into account to ensure a sufficient number of participants could be drawn from each subgroup. A stratified, multi-stage, cluster, sampling design was used to select households within the different areas. From March to September 2013, 7493 individuals from urban and rural areas were invited to participate, of which 5751 agreed (response rate: 76.8%) and provided written informed consent [20]. For participants under the age of 16, parental informed consent was obtained. The study was approved by the ethical committee of the Ministry of Health in Suriname.

### Physical activity assessment

Trained interviewers used the Global physical activity questionnaire (GPAQ) to assess PA [21]. This questionnaire was first translated into Dutch and pre-tested in a small study sample for reliability and face-validity [22,23]. Interviewers asked participants about the number of days and the time they spent doing PA in a typical week within 3 main domains: occupation, transportation and leisure time. Within the occupation domain the activity had to be performed as part of paid or unpaid work, whereas within the transportation domain, PA included walking and/or cycling in order to move from one place to another. Leisure time activities involved all activities that were not included in the occupation or transportation domain. To be considered as an activity, it had to be performed for at least 10 minutes continuously. For activities during occupation and leisure time, participants were asked if it was performed at moderate or vigorous intensity. In order to categorize the activity within the appropriate intensity, examples were given by means of illustrations. Moderate activities were described as activities that cause a small increase in breathing and heart rate, whereas vigorous activities should result in a large increase in breathing and heart rate. For activities within the transportation domain, participants were asked if they walked or cycled at a moderate intensity for at least 10 minutes continuously.

We used the GPAQ analysis guide, provided by the WHO, to clean and analyze the data [24]. According to the guidelines, the data should be excluded if the time spend within a subdomain exceeds 16 hours or participants reported inconsistent or implausible values. Individuals who accumulated throughout a week at least 150 minutes of moderate or vigorous intensity physical activity or an equivalent combination over at least 5 days in the week or 75 minutes of vigorous intensity physical activity on at least 3 days of the week were classified as meeting the recommended PA level. This could be achieved within only one domain or by a combination of the three.

### Socio-demographic information

We divided the country into three areas: the urban coastal area (UCA), the rural coastal area (RCA) and rural interior area (RIA) as defined by the General statistics bureau in Suriname [25]. The RIA is characterized by remote villages within the hinterland, whereas the RCA is less remote, situated near the coast, but does not have all the characteristics of an urban area. Furthermore, participants were categorized based on monthly household income and educational level.

### Ethnicity

For the current analysis we only included the 5 largest ethnic groups: Hindustani, Creole, Maroon, Javanese and mixed ethnicity. The participant’s ethnicity was defined based on self-reported ethnicity of the four grandparents. If at least 3 grandparents were from one ethnic group the participant was classified within that specific ethnic group or else within the mixed ethnicity group [20]. South Asian descendants, mainly from the Indian subcontinent are referred to as the Hindustani population, whereas descendants from Java, Indonesia are referred to as Javanese. For African descendants a distinction was made between descendants that, during slavery remained in the city referred to as the Creole [26], and those that escaped into the hinterland, referred to as Maroons [20,27,28].

### Statistical analysis

In order to have a representation from the Surinamese population, the data were weighted. The weights were calculated to adjust for probability of selection; non-response and differences in age, gender and ethnicity between the sample population and target population. The data from the census 2012 was used as target population [20]. Statistical analyses were performed using SPSS (version 21; SPSS for windows; SPSS Inc, Chicago, IL). Categorical data are presented as numbers (%) and were analyzed using the chi-square test; continuous data as median and mean ± standard deviation (SD).

Unadjusted and adjusted prevalence ratios were calculated for area, gender, age categories and ethnicity, by poisson regression. Because of missing data on income, information of 3255 (72.5%) individuals was included in the regression model. Adjustments were made for area, gender, age categories, level of income and education. A p-value (two-sided) ≤ 0.05 was considered statistically significant.

## Results

Before data cleaning, the study sample included 5751 subjects. Of those, 3 records were not valid and almost 1% of the PA data was missing (n = 57) [20]. Following the GPAQ guidelines [24] we excluded an additional 779 participants for the following reasons: time reported for an activity exceeded 16 hours per day (n = 9); total hours of PA over the 7 days exceeded 168 hours (n = 23); implausible or inconsistent values were reported (n = 747). Data were considered implausible if there was a mismatch between the reported number of days and the time spend active on those days. For instance, the participant reported to be active for a certain period of time, but the reported number of days was zero or missing. Finally, missing data on ethnicity and participants belonging to other groups than the included ethnic groups (n = 428), were excluded , resulting in 4487 valid records.

Table 1 shows the number of subjects and the weighted percentages per area. Overall, the majority 76.0% lived within the UCA with only 15.9% and 8.1% of the population living in the RCA and RIA respectively. Approximately half of the population was male (48.6%) and the average age was 36.2 years (range 15 to 64). There was an equal distribution of men and women (p > 0.05) within and between the three areas; no significant differences were observed regarding age (p > 0.05). However, there were significant differences between the areas with regard to ethnicity with the majority of individuals in the UCA and RCA being of Hindustani origin and the Maroons constituting the largest ethnic group in the RIA. In addition, the economic status and level of education also differed between the areas, whereby a larger proportion of individuals without an income and with no official education lived in the RIA (p < 0.05).

**Table 1:** **Demographic Characteristics of the total population and of the subjects by area**

	**Total population (%)**	**UCA (%)**	**RCA (%)**	**RIA (%)**	**p-value**
Total population	4487 (100)	2312 (76.0)	1482 (15.9)	693 (8.1)	
**Gender**					
Men	1654 (48.6)	882 (48.4)	585 (51.8)	187 (44.5)	0.06
**Age categories**					
Mean age (years)	36.2+/−13.6	36.2+/−13.9	36.9+/−13.6	35.6+/−12.8	
15-24	821 (25.0)	405 (25.3)	248 (24.2)	168 (23.2)	0.53
25-34	1012 (23.5)	494 (23.4)	316 (21.9)	202 (27.3)	
35-44	982 (20.8)	507 (20.3)	347 (22.1)	128 (22.2)	
45-54	981 (19.6)	532 (19.7)	327 (19.9)	122 (17.6)	
55-64	691 (11.2)	374 (11.2)	244 (11.9)	73 (9.7)	
**Ethnic groups**					
Creole	594 (13.0)	351 (14.7)^a^	242 (11.3)^b^	1(0)^c^	<0.001
Hindustani	1195 (33.4)	832 (36.6)^a^	362 (34.9)^a^	1 (0)^b^	
Javanese	842 (16.4)	399 (15.4)^a^	443 (29.6)^b^	0 (0)^c^	
Maroon	1147 (19.3)	267 (13.0)^a^	195 (8.7)^b^	685 (99.5)^c^	
Mix ethnicity	706 (18.0)	463 (20.3) ^a^	240 (15.4)^b^	6 (0.5)^c^	
Economic status*					
No income	390 (6.4)	99 (3.9)^a^	58 (3.2)^a^	233 (36.6)^b^	<0.001
< SRD 800/month	695 (13.4)	295 (12.2)^a^	254 (15.9)^b^	146 (19.9)^b^	
SRD 800-1499/month	1391 (29.1)	683 (28.1)^a^	550 (36.7)^b^	158 (23.4)^a^	
SRD 1500-2199/month	561 (13.2)	329 (13.8)^a^	210 (15.6)^a^	22 (3.8)^b^	
SRD 2200-2899/month	186 (4.7)	155 (5.1)^a^	65 (4.5)^a^	6 (1.1)^b^	
> SRD 2900/month	338 (9.6)	247 (11.2)^a^	78 (5.6)^b^	13 (2.2)^c^	
Education level*					
No education	389 (5.8)	68 (2.7)^a^	44 (2.0)^a^	277 (41.7)^b^	<0.0001
Low level	3234 (71.5)	1613 (70.4)^a^	1225 (85.1)^b^	396 (55.1)^c^	
High level	805 (21.5)	597 (25.6)^a^	196 (11.9)^b^	12 (1.6)^c^	
**Meeting recommended level of PA**					
Moderate-vigorous PA level	2377 (55.5)	1222 (55.7)^a^	816 (57.9)^a^	339 (49.1)^b^	0.02

Abbreviations: UCA: urban coastal area, RCA: rural coastal area ; RIA: rural interior area; SRD = Surinamese Dollar. The percentages between brackets are weighted percentages of the total population within that area. ^a^ significant different from ^b^ and ^c^at a level of P < 0.05; ^b^ significant different from ^a^ and ^c^ at a level of P < 0.05; ^c^ significant different from ^a^ and ^b^at a level of P < 0.005. * The numbers do not add up to the column total because of missing data on income and education level.

Regarding PA, 55.5% (n = 2377) of the total population met the WHO recommendations. Further, compared to individuals from the UCA, individuals living within the RIA were less likely to meet the recommended PA levels, whereas the prevalence of meeting these recommendations was higher for individuals living in the RCA (p for trend <0.02) (Table 2).

**Table 2:** **Percentage and unadjusted and adjusted prevalence ratios for meeting the recommended PA level**

	**Weighted %**	**Unadjusted PR (CI)**	**Adjusted PR (CI)**
**Area** ^**a**^			
Urban coastal	*55.7*	*1 (reference)*	*1 (reference)*
Rural coastal	*57.9*	*1.09 (1.01-1.17)*	*1.06 (0.99-1.15)*
Rural interior	*49.1*	*0.86 (0.77-0.97)*	*0.86 (0.74-0.99)*
**Gender** ^**b**^			
Male	*62.4*	*1 (reference)*	*1 (reference)*
Female	*49.0*	*0.77 (0.73-0.82)*	*0.78 (0.74-0.84)*
**Age categories** ^**c**^			
15-24	*62.7*	*1 (reference)*	*1 (reference)*
25-34	*54.7*	*0.86 (0.79-0.93)*	*0.87 (0.80-0.95)*
35-44	*56.5*	*0.89 (0.82-0.97)*	*0.91 (0.83-0.99)*
45-54	*53.8*	*0.85 (0.78-0.93)*	*0.85 (0.78-0.93)*
55-64	*42.6*	*0.69 (0.61-0.79)*	*0.71 (0.62-0.81)*
**Ethnicity** ^**d**^			
Hindustani	*56.6*	*1 (reference)*	*1 (reference)*
Maroon	*53.4*	*0.91 (0.83-0.99)*	*1.02 (0.91-1.14)*
Mix ethnicity	*56*	*0.96 (0.87-1.05)*	*0.96 (0.88-1.05)*
Javanese	*50.3*	*0.93 (0.85-1.02)*	*0.92 (0.84-1.00)*
Creole	*61.6*	*1.07 (0.98-1.17)*	*1.07 (0.98-1.17)*
**Economic status** ^**e**^			
No income	*33.9*	*1 (reference)*	*1 (reference)*
< SRD 800/month	*52.9*	*1.59 (1.34-1.89)*	*1.58 (1.33-1.87)*
SRD 800-1499/month	*61.6*	*1.76 (1.49-2.05)*	*1.71 (1.45-2.02)*
SRD 1500-2199/month	*57.4*	*1.64 (1.39-1.95)*	*1.61 (1.35-1.92)*
SRD 2200-2899/month	*57.9*	*1.68 (1.38-2.04)*	*1.63 (1.33-1.98)*
> SRD 2900/month	*52.5*	*1.50 (1.26-1.80)*	*1.46 (1.21-1.75)*
**Education level** ^**f**^			
No education	*38.6*	*1 (reference)*	*1 (reference)*
Low level	*57.5*	*1.51 (1.28-1.77)*	*1.40 (1.18-1.66)*
High level	*53.6*	*1.38 (1.16-1.64)*	*1.32 (1.10-1.58)*

The weighted percentages are calculated for the 4487 subjects, whereas the prevalence ratio analysis is based on 3255 subjects because of missing data for the economic status. PR = Prevalence ratio ; CI = Confidence interval

^a^Adjusted for gender, age category and ethnicity.

^b^Adjusted for area, age category and ethnicity.

^c^Adjusted for gender, area and ethnicity.

^d^Adjusted for area, gender and age category.

^e^Adjusted for area, gender, age category and ethnicity.

^f^Adjusted for area, gender, age category and ethnicity.

Recommended PA level conform the World health organization guidelines.

**Figure 1:**
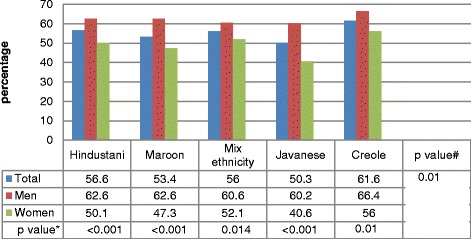
Percentage of total population, men and women meeting the recommended PA level per ethnic group. Legend: #: significance level for difference between ethnic groups;* significance level for difference between men and women*.*

As shown in Table 2, women were always less likely to meet the recommended PA levels compared to men (p for trend < 0.0001). This significant difference was seen across all ethnic groups (Figure 1). Likewise, the prevalence ratio’s decreased with increasing age from 62.7 in the younger individuals to 42.6 in the oldest participants (p for trend <0.0001). With 61.6%, the Creole population had the highest percentage of individuals meeting the recommended PA level (p =0.001), whereas the lowest percentage was reported by the Javanese population. Finally, the lowest percentage of participants meeting the recommend PA level was found in the group with no household income and no formal education. According to the guidelines, the PA recommendations can be met by being active only within one domain or by being active within two or three domains combined. Table 3 depicts the quartile values for the percentage that one domain contributes to the overall PA of the participants that met the recommendations. Within all areas the working domain contributes for the most part to the overall PA, though the median value is the highest within the RIA (86.8%), followed by the RCA (73.6%). Table 3 also shows that leisure time activity has a low contribution to the overall PA, with the 75^th^ percentile at 28% for the overall population and at 34.5%, 13% and 1.3% for the UCA, RCA and IRA respectively. In all ethnic groups, the working domain contributed the most to the total PA. The highest median value was found for the Maroon population 76.9%, whereas the lowest value was found for the mixed ethnicity group 49.4%. For these two ethnic groups the 75^th^ percentile for leisure time PA was respectively 6.8% and 44.4% which were the lowest and highest reported values. With a median value of 7.7% the transport domain has the lowest contribution to the overall PA level for the Javanese population that meets the recommended level of PA (Table 3)

**Table 3:** **Contribution of the three domains to the overall physical activity per area and ethnicity**

			**25th quartile**	**Median**	**75th quartile**
**Total population**		Work	4.7	63.6	94.1
		Transport	0	12.7	45.5
		Leisure time	0	0	28
**Area**	**Work**	UCA	0	57.1	93.2
		RCA	14.6	73.6	94.8
		RIA	66.7	86.8	98.2
	**Transport**	UCA	0	13.3	48.3
		RCA	0	14.3	45.6
		RIA	0	10	24.3
	**Leisure time**	UCA	0	0	34.5
		RCA	0	0	13
		RIA	0	0	1.3
**Ethnicity**	**Work**	Hindustani	6.4	66.6	95.8
		Maroon	9.0	76.9	95.8
		Mix ethnicity	0	49.4	86.9
		Javanese	22.2	72.6	96.3
		Creole	3.3	53.4	88.3
	**Transport**	Hindustani	0	11.1	44.4
		Maroon	0	13.0	36.3
		Mix ethnicity	0	20	50
		Javanese	0	6.9	42.6
		Creole	1.1	19.5	46.6
	**Leisure time**	Hindustani	0	0	23.4
		Maroon	0	0	6.8
		Mix ethnicity	0	0	44.4
		Javanese	0	0	25.6
		Creole	0	0	36.3

The values are the percentages that the working, transport or leisure time domain contributes to the overall physical activity. The median value, the first and third quartiles are given for the total population, per area and ethnicity. UCA = urban coastal area, RCA = rural coastal area, RIA = rural interior area.

.

## Discussion

Our study showed that within a representative multi-ethnic population of Suriname, only 55.5% of the population met the recommended PA levels based on the WHO guidelines. In general, women were less active compared to men and the proportion of individuals meeting the recommendations decreased with increasing age, even after adjusting for important covariates. Further, people living in the RIA were less likely to meet the recommended PA levels. When comparing the ethnic groups, the lowest percentage of individuals meeting the recommendations was within the Javanese population.

Our findings are in line with data from the Caribbean region, where between 51.9% - 74.5% of the population met the recommended PA level [29] and are somewhat lower than data from the American region, where, irrespective of gender, 56.7% of the population met the recommended PA level [7]. Guthold et al. also compared PA data from 4 South American countries (Brazil, Ecuador, Paraguay and Uruguay) and reported that between 64.6% - 82% of men and between 69.8% - 79.1% of women met the recommendations [13]. Compared to the data from Guthold et al., our findings for men are within the range and for women are lower than the reported range. As noted from these studies there exists a great variance in PA behavior between countries and between different regions [7,12,13]. This could be attributed to several factors including the use of different questionnaires. Guthold et al. used the short form of the international physical activity questionnaire (IPAQ) [13], whereas other studies used the long form of IPAQ or the GPAQ [7,29].

The GPAQ, used in our study, has previously been used in the African region, India and the Czech Republic [6,14,30,31]. Compared to these countries the Surinamese population seems less active than those in the African region or in the Czech Republic but scores better compared to India. This variance could be explained by several other factors including the age categories selected within the studies [13]. The participants selected in respectively Czech republic, the African region and India were between 18–90 years, 24–69 years and 20 years and older. Also cultural differences in interpreting the questionnaire, and the inclusion of different areas could influence the variance [19,31], so comparisons require caution.

Comparing different areas some studies reported a higher prevalence of PA within the rural area than the urban area [13,14,32], whereas others found the opposite [19]. Our results showed that, compared to the UCA, the proportion of people in the RIA meeting the recommended PA level is lower (p < 0.05), whereas the proportion of people in the RCA is higher (p > 0.05). This difference could be attributed to a difference in economic status and/or education level, since these covariates have been shown to be associated with PA [33-35]. Within our population the economic status was significantly different between the 3 areas, where the proportion of people within almost every income category differed between the areas. This was also seen for the education level, where 41.7% of the RIA population had no formal education level. However some caution might be warranted as a large proportion of the population (33%) did not give information on their economic status and education level. Even though, Dutch is the official language in Suriname and in school, only 5.7% of the RIA population (80.3% of the total population) was interviewed in Dutch. As the interviewers were also fluent in speaking the native languages of the RIA, they translated the questionnaire when needed. In case further clarification of a question was needed, showing cards were used. Furthermore, there is also a significant difference between the areas for the ethnic groups, where 99% of the Maroon population lives in the RIA. These covariates might have influenced the difference found between areas and further investigation is needed.

In agreement with others, [5,6,12,13,30,31,36] we observed that a lower proportion of women met the recommended PA level (p < 0.05), which remained significant after adjusting for covariates. Comparison of ethnic groups showed that the lowest percentage of women meeting the recommended PA level was found within the Javanese population. Further, older people were always less likely to meet the recommended levels of PA which is also a consistent finding in other populations [6,34].

Cardiovascular disease mortality data in Suriname shows that, Hindustani’s account for 33.7% of all cardiovascular deaths, followed by the Creole population (28%) [37]. This difference in mortality between ethnic populations can partly be explained by genetic variations [38] but most likely also by differences in lifestyle [39]. Therefore, in a multi-ethnic population, like in Suriname, an adequate evaluation of PA behavior in relation to the ethnic and cultural differences is needed. In our study, a lower proportion of Hindustani’s met the recommended PA level compared to the Creole population, which was similar to results from The Netherlands [11]. Furthermore, we found the lowest proportion of individuals being sufficiently active among the Javanese population (p = 0.07). Unfortunately, to the best of our knowledge, there are no data available on the prevalence of PA among Javanese communities outside Suriname which makes comparisons difficult. In order to develop effective PA intervention progams it is important to take these ethnic and cultural differences in PA into account [40].

For the development of PA intervention programs, it is furthermore recommended to investigate the different domains of PA, since different associations have been found for each of the separate domains with CVD mortality [41]. Prior studies conducted in India [32] and Mozambique [31], reported that in the rural areas , the working domain contributed more to the overall PA. That is, individuals living in the rural areas were more physically active in the working domain compared to the urban regios [31]. Our results also showed that the working domain had a bigger contribution to the overall PA level within the RIA compared to the two other areas. Since 99% of our population within the RIA is made up of the Maroon ethnic group, this high contribution of the working domain to the overall PA is also seen within this ethnic group. Within the RIA there is still a need for physically demanding labour and less time for leisure time activities compared to the fast growing UCA, where there are more sedentary working activities and increasing use of motorized transportation. With a very low contribution of the leisure time domain to the PA level for the total population and since participation in leisure time PA has been associated with an increase in life expectancy [42] it is important to focus on interventions to increase leisure time PA.

Our study has some limitations that need to be taken into consideration. First, the results are based on self-reported information which is sensitive for overestimation or recall bias [43-45]. Furthermore, since being physically active is a socially preferable activity, the use of questionnaires may lead to social desirability bias [43,45]. In order to identify if the observed differences in meeting the recommended PA level between the areas, ethnic groups and age categories in our study is not only based on the administration of the questionnaire and its inherent limitations, objective measurement of PA might be warranted in future research. Several objective and relatively affordable activity monitors have been identified and have been shown to be reliable and valid in measuring PA behavior [43]. However, also these objective measurements have their limitations that need to be taken into consideration when using these tools for future research for PA surveillance [46].

Secondly, seasonal variations have been reported to influence PA behavior [43]. Suriname is located in the tropical region of South America and experiences only small seasonal variations, which are mainly characterized by differences in rainfall and not by temperature or humidity. Hence, based on the distribution of rainfalls 4 seasons are identified for Suriname. These are: (a) the short wet season (beginning of December – beginning of February), (b) the short dry season (beginning of February – end of March), (c) the long wet season (beginning of April – mid of August), (d) the long dry seasons (mid of August – end of November) [47]. Half of the recruitment period (3.5 months) involved the wet season (May – mid of August) and the other half (3.5 months) was performed during the large dry season (mid of August – September) and the short dry season (March – April). Therefore, as recruitment was equally balanced between the wet and dry seasons, we believe that seasonality will not have had a major impact on our overall results. Nevertheless, given these differences in rainfall, future research should investigate whether PA behavior differs according to seasonality in Suriname.

Finally, in our study, the validation of the translated GPAQ was based on the face-validation [22,23] procedure whereby, as part of a pilot study, a small sample of participants was interviewed by a trained interviewer. The results were reviewed by a panel of experts*.* Further, Bull et al. showed earlier that overall the GPAQ provides reproducible data and showed a moderate – strong positive correlation with IPAQ and a poor to fair correlation for criterion validation procedure in low and middle income countries. Nevertheless, they concluded that the GPAQ is a suitable and acceptable instrument for monitoring physical activity in population health surveillance systems [21].

## Conclusion

Approximately half of the Surinamese population meets the recommended PA level. A significant difference between men and women and also between people living in the RIA compared to the other areas is observed. Also within our population the proportion of subjects being physically active decreased with increasing age and the lowest percentage of PA is observed in the Javanese population. To our knowledge this is the first nationwide study performed in Suriname investigating the PA behavior and allowing an appropriate comparison between the areas, gender, age categories and ethnic groups. The obtained result can be used to identify groups and/or regions within the Surinamese population that need more attention or a different way of handling when designing or implementing PA intervention programs as part of a strategy to reduce the NCD burden.

## Abbreviations

CVD: Cardiovascular disease
GPAQ: Global physical activity questionnaire
IPAQ: International physical activity questionnaire
NCD: noncommunicable disease
PA: Physical activity
RCA: Rural coastal area
RIA: Rural interior area
STEPS: WHO STEPwise approach
UCA: Urban coastal area
WHO: World Health Organization
